# Bearing Dynamics Modeling Based on the Virtual State-Space and Hammerstein–Wiener Model

**DOI:** 10.3390/s24165410

**Published:** 2024-08-21

**Authors:** Genghong Jiang, Kai Zhou, Zhaorong Li, Jianping Yan

**Affiliations:** 1 Zhejiang Institute of Communications, Hangzhou 311112, China; jghong@zjvtit.edu.cn; 2 School of Aeronautics and Astronautics, Zhejiang University, Hangzhou 310027, China; kinozhou@zju.edu.cn

**Keywords:** bearing, Hammerstein–Wiener, data-driven, dynamic modeling, Prognostics and Health Management

## Abstract

This study investigates a novel approach for assessing the health status of rotating machinery transmission systems by analyzing the dynamic degradation of bearings. The proposed method generates multi-dimensional data by creating virtual states and constructs a multi-dimensional model using virtual state-space in conjunction with mechanism model analysis. Innovatively, the Hammerstein–Wiener (HW) modeling technique from control theory is applied to identify these dynamic multi-dimensional models. The modeling experiments are performed, focusing on the model’s input and output types, the selection of nonlinear module estimators, the configuration of linear module transfer functions, and condition transfer. Dynamic degradation response signals are generated, and the method is validated using four widely recognized databases consisting of accurate measurement signals collected by vibration sensors. Experimental results demonstrated that the model achieved a modeling accuracy of 99% for multiple bearings under various conditions. The effectiveness of this dynamic modeling method is further confirmed through comparative experimental data and signal images. This approach offers a novel reference for evaluating the health status of transmission systems.

## 1. Introduction

The mechanical transmission system plays an indispensable key role in transportation. For example, over 45% of maritime machinery failures are caused by transmission systems. Therefore, developing intelligent operation and maintenance technologies for transmission systems is imperative. Prognostics and Health Management (PHM) has emerged as a valuable strategy for preventing failures and reducing costs, garnering increasing research interest. Bearing failure, which accounts for over 50% of mechanical failures, can be a significant research object. This research aims to provide new methods of fault modeling and ideas for developing health management technology for transmission systems.

**Figure 1:**
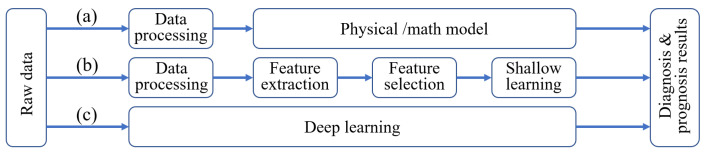
Modeling methods in PHM: (**a**) mathematical and physical models, (**b**) shallow learning, (**c**) deep learning.

In PHM, fault modeling techniques are categorized into mechanism-based and data-driven methods. Mechanism-based methods employ mathematical and physical models, as illustrated in method (a) in Figure 1 [1]. Bearing degradation is typically characterized using vibration and temperature sensors, with vibration signals from acceleration sensors being particularly prevalent due to their sensitivity and rapid response. A significant amount of research in bearing PHM has focused on mechanism modeling [2,3,4]. Notable advances include dynamic models based on Hertz contact theory and finite element methods for detecting defects in angular contact ball bearings [5], as well as more complex models incorporating local geometrical imperfections and varying race-supporting conditions [6]. Qian et al. [7] developed a model-based fault diagnosis technology for rotor-bearing systems, effectively utilizing residual generation techniques for fault detection and localization.

Despite these advancements, the application of mechanism-based methods in industrial settings is limited due to noisy and complex operating environments. Consequently, data-driven methods are increasingly prominent in bearing PHM research. These methods leverage machine learning techniques to capture dynamic degradation characteristics from measurement data and produce high-precision models. These techniques include shallow and deep learning techniques, as depicted in methods (b) and (c) in Figure 1. Since Janssens et al. first applied Convolutional Neural Networks (CNNs) to bearing fault diagnosis in 2016 [8], various enhancements have been proposed, such as 2D-CNN, multiscale CNN, and adaptive CNN [9,10,11]. Other techniques like Long Short-Term Memory (LSTM), Deep Belief Networks (DBN), Recurrent Neural Networks (RNN), and Deep Deterministic Policy Gradient (DDPG) algorithms have also demonstrated efficacy in fault diagnosis and modeling [12,13,14,15,16]. For instance, Tian et al. proposed a CNN-LSTM fault diagnosis model optimized using Hybrid Particle Swarm Optimization (HPSO), achieving a classification accuracy of 99.2% [17]. However, data-driven modeling methods face challenges, such as the need for extensive training datasets and a lack of interpretability, particularly in hyperparameter optimization.

To address these challenges, this study proposes a bearing dynamics modeling method utilizing virtual state-space and Hammerstein–Wiener (HW) models. This approach generates the necessary data for modeling by constructing multi-dimensional virtual states. It enhances model interpretability through the HW model’s capacity to capture nonlinear system characteristics, thereby improving modeling accuracy. Although HW models are predominantly studied in the control field [18,19], their robust data fitting capabilities for nonlinear systems suggest the potential for high-precision dynamic modeling of bearing degradation and anomalies. Additionally, the HW model’s modular structure offers enhanced configurability and extensibility. For example, Wang et al. integrated HW models with neural networks, achieving lower average absolute percentage errors (0.0223) compared to Gated Recurrent Units (GRU) and Recurrent Neural Networks (RNN) on simulation data [18]. Zhong et al. [19] explored parameter estimation for HW time-varying systems using a learning recognition algorithm with a forgetting factor. Based on these advancements, this study combines the HW model with virtual state-space for fault modeling in PHM, validating it through dynamic modeling of bearings and providing a novel approach for assessing transmission system health. The innovations of this study include the following:

1. This study uses multi-dimensional virtual state-spaces to generate sufficient data, improving the accuracy of model identification.
2. This study constructs multi-dimensional models of the dynamic system by utilizing the virtual state-spaces, which allows for high-precision modeling, mainly through the dimension with a more vital characterization ability of the dynamic system.
3. This study employs the HW model in the control field to effectively mitigate the challenges posed by the nonlinear part of the system in dynamic modeling.

**Figure 2:**
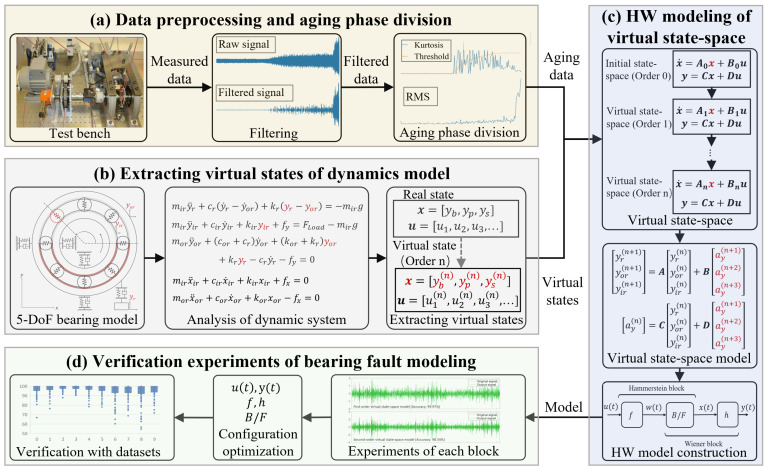
Research structure.

Figure 2 illustrates the research structure, comprising four components: (a) methods for obtaining high-quality signals and selecting valid data; (b) extraction of virtual states for fault modeling through dynamic analysis; (c) application of virtual state-space to bearing fault modeling and HW method for model identification; and (d) verification experiments of the proposed method. The paper is organized as follows: Section 2 covers theoretical foundations, Section 3 describes the database and data processing, Section 4 presents experimental results for modeling configuration, Section 5 validates the optimal configuration and method with various datasets, and Section 6 concludes the study.

## 2. Theoretical Foundation

### 2.1. Bearing 5-DoF Model for Dynamics Analysis

**Figure 3:**
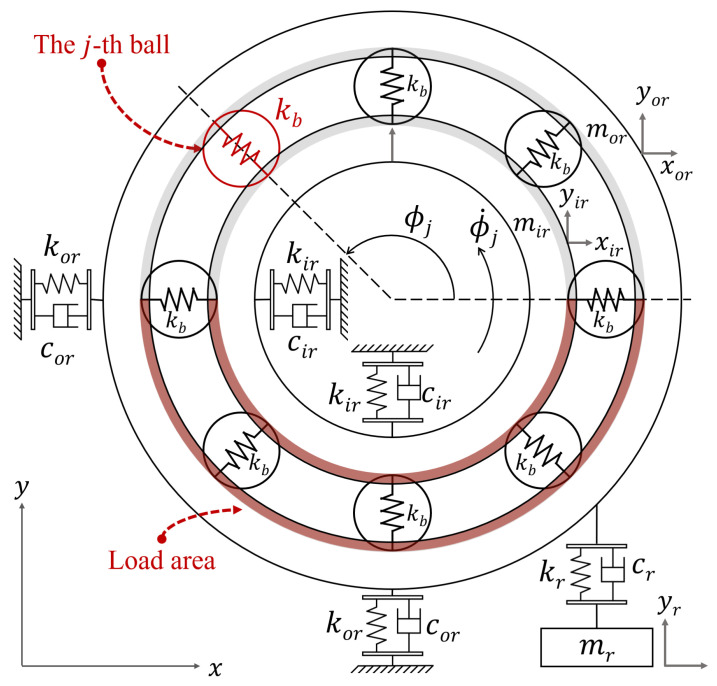
5-DoF model of bearing.

This study uses bearings as a case study to investigate a new fault modeling method. The foundational analysis employs the 5-DoF model, central to subsequent research. This model, shown in Figure 3, is mathematically represented by Equations (1)–(5) [20]:

(1) $$\begin{array}{ccc} & & m_{ir}{\ddot{x}}_{ir}+c_{ir}{\dot{x}}_{ir}+k_{ir}x_{ir}+f_{x}=0, \end{array}$$

(2) $$\begin{array}{ccc} & & m_{ir}{\ddot{y}}_{ir}+c_{ir}{\dot{y}}_{ir}+k_{ir}y_{ir}+f_{y}=F_{load}-m_{ir}g, \end{array}$$

(3) $$\begin{array}{ccc} & & m_{or}{\ddot{x}}_{or}+c_{or}{\dot{x}}_{or}+k_{or}x_{or}-f_{x}=0, \end{array}$$

(4) $$\begin{array}{ccc} & & m_{or}{\ddot{y}}_{or}+\left(c_{or}+c_{r}\right){\dot{y}}_{or}+\left(k_{or}+k_{r}\right)y_{or}-k_{r}y_{r}-c_{r}{\dot{y}}_{r}-f_{y}=0, \end{array}$$

(5) $$\begin{array}{ccc} & & m_{r}{\ddot{y}}_{r}+c_{r}\left({\dot{y}}_{r}-{\dot{y}}_{or}\right)+k_{r}\left(y_{r}-y_{or}\right)=-m_{r}g, \end{array}$$

where $m_{ir}$, $m_{or}$, $m_{r}$, respectively, represent the mass of the inner race, outer race, and the unit resonator; $k_{ir}$, $k_{or}$, $k_{r}$, $c_{ir}$, $c_{or}$, $c_{r}$ represent their stiffness and damping, respectively; $f_{x}$, $f_{y}$ represent the nonlinear contact force of the bearing in the horizontal and vertical direction, respectively; $x_{ir}$, $y_{ir}$, $x_{or}$, $y_{or}$, $y_{r}$ mean the two inner and outer race DoF, and measured vibration response, respectively. Additionally, *g* represents the gravitational acceleration, and $F_{load}$ denotes the vertical external force applied to the inner race of the bearing [21].

The system is simplified using the lumped parameter method by treating all balls as a single entity. The contact force $f_{j}$ between the *j*-th ball and the raceway is split into horizontal and vertical components ($f_{x_{j}}$ and $f_{y_{j}}$). Then, through Newton’s second law, the vertical acceleration $a_{y}$ and can be given by Equation (6) [3]:

(6) $$a_{y}=\frac{f_{y}}{m}=\frac{\sum_{j=1}^{n_{b}}f_{y_{j}}}{\sum_{j=1}^{n_{b}}m_{j}},$$

where $n_{b}$ denotes the number of balls in the bearing and $m_{j}$ the mass of the *j*-th ball. From the Hertz contact theory, the contact force $f_{j}$ and its vertical component $f_{y_{j}}$ between the *j*-th ball and the raceway are given by Equations (7) and (8), respectively:

(7) $$f_{j}=k_{b}\delta_{j}^{1.5},$$

(8) $$f_{y}=k_{b}\sum_{j=1}^{n_{b}}\gamma_{j}\delta_{j}^{1.5}\sin\phi_{j},$$

where $k_{b}$ represents the stiffness of the balls. $\gamma_{j}$ is a switch function indicating the ball is within the load region, thereby enabling deformation due to the contact force $f_{j}$. The total deformation $\delta_{j}$ of the *j*-th ball is related to the relative displacement between inner and outer races, its angular position $\phi_{j}$, and the bearing clearance. $\gamma_{j}$ and $\delta_{j}$ are calculated by Equations (9) and (10), respectively [3]:

(9) $$\gamma_{j}=\left\{\begin{array}{c} 1,\;\delta_{j}>0,\\ 0,\;\mathrm{otherweise}, \end{array}\right.$$

(10) $$\delta_{j}=\left(x_{ir}-x_{or}\right)\cos\phi_{j}+\left(y_{ir}-y_{or}\right)\sin\phi_{j}-c,$$

where *c* stands for the clearance. Many essential state quantities in the dynamic model are difficult to measure in practice, such as $(y_{ir}$ and $y_{or}$) in Figure 3, which makes it difficult to establish an accurate physical model. Therefore, this study will explore constructing a high-precision fault model based on dynamic analysis from the perspective of acceleration response and multi-dimensional data.

### 2.2. Virtual State-Space for Dynamics Modeling

This study’s theoretical foundation is based on the concept of virtual states and the virtual state-space model for generating multi-order data and exploring system dynamic characteristics from multiple dimensions [22]. The structural and compositional expressions of the virtual state-space align with the general state-space formulation, as illustrated in Equations (11) and (12):

(11) $$\begin{array}{ccc} {\dot{x}}_{v} & = & Ax_{v}+Bu_{v}, \end{array}$$

(12) $$\begin{array}{ccc} y_{v} & = & Cx_{v}+Du_{v}. \end{array}$$

where the state matrix A, input matrix B, output matrix C, and feed-forward matrix D retain their standard definitions and functions. The primary distinction lies in the virtual states within the virtual state vector $x_{v}$. These virtual states possess undefined but real physical significance, which can be represented by one or more derivatives of the actual state. Consequently, the variables in the virtual output vector $y_{v}$ and virtual input vector $u_{v}$ can also be obtained by derivation of actual measurable variables.

Based on the relationships between various actual state variables in the dynamic model, $y_{ir}$, $y_{or}$, and $y_{r}$ can be selected as the actual state variables in the initial bearing model (0th-order). The derivatives of these states retain specific physical significance, fulfilling the requirements of the theory of virtual states. Additionally, the vertical acceleration signal ${\ddot{y}}_{r}$ is a measured physical variable associated with the state variables $y_{ir}$, $y_{or}$ and $y_{r}$. Therefore, ${\ddot{y}}_{r}$ can be used as an in- or output of the initial model and expressed as $a_{y}={\ddot{y}}_{r}$. As an example of a three-input single-output model, Equations (13) and (14) can be used to construct the initial and virtual state-space models for the bearing:

(13) $$\begin{array}{ccc} \left[\begin{array}{c} {\dot{y}}_{r}\\ {\dot{y}}_{or}\\ {\dot{y}}_{ir} \end{array}\right] & = & A\left[\begin{array}{c} y_{r}\\ y_{or}\\ y_{ir} \end{array}\right]+{\mathrm{Bu}}_{v}, \end{array}$$

(14) $$\begin{array}{ccc} a_{y} & = & C\left[\begin{array}{c} y_{r}\\ y_{or}\\ y_{ir} \end{array}\right]+{\mathrm{Du}}_{v}. \end{array}$$

**Figure 4:**
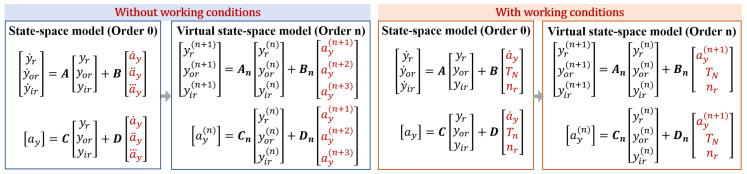
Multi-order virtual state-space model.

Higher-order virtual state-spaces can be derived from the initial state-space using different input forms, as depicted in Figure 4. Multi-order virtual state-space models can also be developed through high-order derivatives of initial inputs and outputs, providing a data foundation for model identification. In virtual state-space equations consisting of various orders, variations in the state matrix $A_{n}$ reflect the degradation of bearing dynamics in multiple dimensions, with specific parameters that may contain information regarding bearing aging and faults. Thus, virtual state-spaces serve two primary purposes: generating extensive multi-dimensional data from limited data and exploring system dynamic degradation from various dimensions to uncover implicit dynamic characteristics.

Based on the inference of generating multi-dimensional data, multiple-order derivatives of the acceleration signal $a_{y}$ can serve as in- and outputs for the virtual state-space model. Hence, the in- and output vectors can include one or more of the acceleration signal $a_{y}$ and its multiple-order derivatives $a_{y}^{\left(i\right)}$, where i=1,2,3,…,n, ($n\in N^{+}$). Although the time function of $a_{y}$ is assumed to be unknown during model identification, high-frequency sensors provide the minimum interval between sampling points. This approach allows for the use of finite difference rather than differentiation as an alternative method to generating multi-dimensional data.

Low-order derivatives (e.g., velocity and acceleration) of physical variables (e.g., displacement) are typically well-defined and measurable, making them suitable for input or output in virtual state-space models. However, higher-order derivatives are more challenging to obtain. Therefore, finite difference can be employed in place of differentiation to calculate derivatives in specific scenarios, such as sampling at very high frequency. The minimal time intervals (Δt≤0.0001 s) between points $t_{0}$ and $t_{0}+\Delta t$ make it justifiable to replace differentiation with finite differences, as represented in Equation (15):

(15) $${\dot{a}}_{y}=\frac{da_{y}}{dt}\approx \frac{\Delta a_{y}}{\Delta t}=\frac{a_{y}\left(t_{0}+\Delta t\right)-a_{y}\left(t_{0}\right)}{\Delta t}.$$

**Figure 5:**
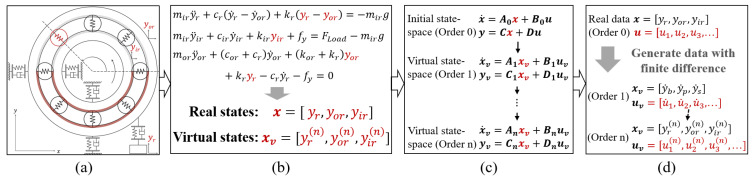
Construction process of virtual state and virtual state-space: (**a**) 5-DoF model, (**b**) dynamics analysis, (**c**) virtual state generation, (**d**) virtual state-space construction.

Simultaneously, filtering the generated data can help mitigate the adverse effects of using finite differences to calculate derivatives. The entire workflow for utilizing virtual state-space in bearing fault modeling is depicted in Figure 5.

### 2.3. Hammerstein–Wiener Method for Model Identification

Based on the framework of multi-dimensional virtual state-space, this study proceeds to identify dynamic systems with known inputs and outputs. Given the complexities of bearings in real-world applications and the inherent nonlinearity of their acceleration signal fluctuations, the HW modeling method is employed. While HW models have demonstrated significant success in various nonlinear systems and control applications [23], their application in PHM is relatively underexplored. Thus, a key innovation of this study is integrating the HW model with virtual state-space, which facilitates high-precision model identification for multi-dimensional bearing dynamics and explores new methods for assessing the health of transmission systems.

**Figure 6:**
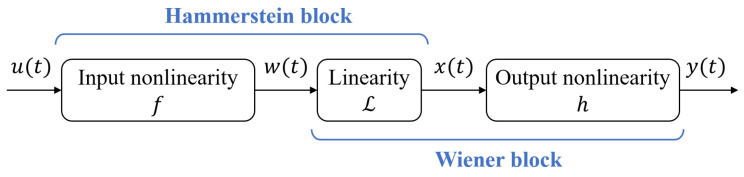
HW model structure.

The HW model can serve as a black-box for estimating linear models, with its accuracy enhanced by incorporating nonlinearities in the inputs and outputs. It can also function as a grey-box model that captures physical process characteristics. As illustrated in Figure 6, the HW model simplifies a complex nonlinear dynamic system into two static nonlinear blocks, *f* and *h*, in series with a linear block L, where u(t) and y(t) represent the input and output data, respectively. The the linear block’s input and output are denoted as w(t) and x(t). The model structure is mathematically described by Equations (16)–(18):

(16) $$\begin{array}{ccc} w(t) & = & f(u(t),\alpha ), \end{array}$$

(17) $$\begin{array}{ccc} y(t) & = & h(x(t),\beta ), \end{array}$$

(18) $$\begin{array}{ccc} x(t) & = & L(w(t),\vartheta ), \end{array}$$

where α, β, and ϑ ($\alpha \in R^{n_{\alpha }}$, $\beta \in R^{n_{\beta }}$, $\vartheta \in R^{n_{\vartheta }}$) are parameter vectors of memoryless nonlinearities *f* and *h*, and the linear time-invariant system L. The HW model represents the dynamics of the polynomial-based linear block L within the system using the discrete transfer function H(s) in Equation (19) [24]:

(19) $$H\left(s\right)=\frac{x(t)}{w(t)}=\frac{B\Pi_{N=1}^{N_{Z}}\left(s-z_{n}\right)\left(s-z_{n}^{*}\right)}{\Pi_{k=1}^{N_{p}}\left(s-p_{k}\right)\left(s-p_{k}^{*}\right)},$$

where $N_{Z}$ and $N_{p}$ represent the number of zero poles, respectively, $z_{n}$, $z_{n}^{*}$, $p_{k}$ and $p_{k}^{*}$ denote the values of zeros and poles along with their complex conjugates, and *B* represents the scaling coefficient. For instance, in a two-input-two-output system, the specific Hammerstein nonlinearity *f* and Wiener nonlinearity *h* are given by Equations (20) and (21) [25]:

(20) $$f\left(u\left(t\right),\alpha \right)=\left[\begin{array}{c} f_{1}\left(u\left(t,1\right),\alpha \right)\\ f_{2}\left(u\left(t,2\right),\alpha \right) \end{array}\right],$$

(21) $$h\left(u\left(t\right),\beta \right)=\left[\begin{array}{c} h_{1}\left(u\left(t,1\right),\beta \right)\\ h_{2}\left(u\left(t,2\right),\beta \right) \end{array}\right],$$

where $f_{1}$ and $h_{1}$ represent the input and output nonlinearities corresponding to the first input u(t,1), while $f_{2}$ and $h_{2}$ correspond to those associated with the second input u(t,2). This block structure provides strong data fitting capabilities, reconfigurability, and parameter interpretability. The HW model does not require complete identification of the nonlinear process; instead, it uses nonlinear functions *f* and *h* to process the linear system’s input and output signals, effectively capturing the system’s nonlinear characteristics.

In the modeling experiment, attention is given to the stability, accuracy, and efficiency of configuring the nonlinear functions *f* and *h*. Firstly, the nonlinear functions must avoid introducing mathematical errors to ensure process stability. Secondly, high accuracy is crucial to reflect the model’s fitting capability. Lastly, the modeling process should balance stability and accuracy to achieve efficiency. The dynamic components of the linear module involve three key parameters: input delay, and number of zeros and poles. Input delay influences the system’s response to external stimuli over time, while the quantity and values of zeros and poles affect the stability and performance of the dynamic system.

## 3. Database and Data Preprocessing

**Figure 7:**
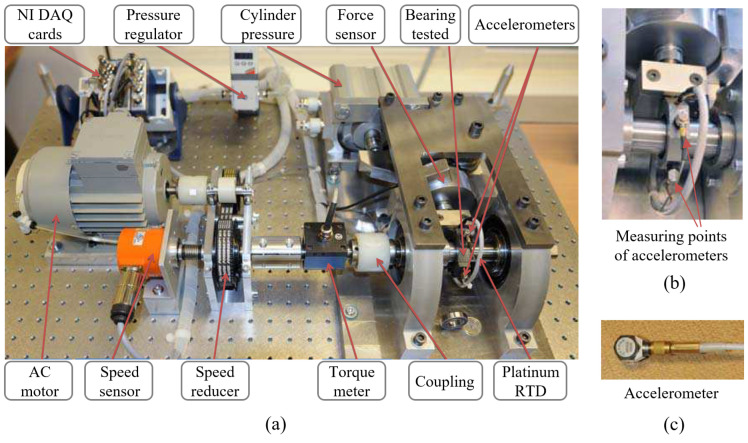
Test bench and sensors: (**a**) the PRONOSTIA platform, (**b**) measuring points in vertical and horizontal directions, (**c**) accelerometer.

The proposed method is evaluated using the IEEE PHM 2012 Challenge dataset, a widely recognized resource in the field of PHM. This dataset’s rolling bearing test data are collected from the PRONOSTIA experimental platform at the FEMTO-ST research institute and have been previously used in the IEEE PHM 2012 Prognostics Challenge. The PRONOSTIA platform and accelerometers are shown in Figure 7 [26].

**Figure 8:**
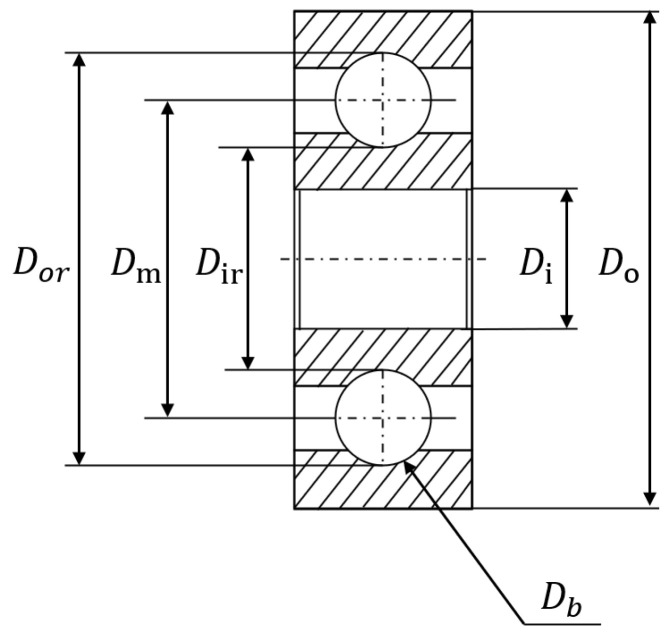
Bearing cross-section.

In the measurement part of the test bench, various high-quality sensors are used to obtain the instantaneous radial force applied to the bearing, the speed of the shaft handling the bearing, and the torque applied to the bearing. The acceleration sensors (Type DYTRAN 3035B) operate in vertical and horizontal directions at a sampling frequency of 25.6 kHz. Additionally, this sensor collects samples for 0.1 s every ten seconds. The test bearing is illustrated in Figure 8, and its parameters are listed in Table 1.

Bearing accelerated degradation experiments are conducted until the vibration signal amplitude exceeds 20 g. As detailed in Table 2, these run-to-failure datasets include 17 rolling bearings tested under three different operating conditions. The operating conditions in the experiments are set to higher levels than typical industrial scenarios to expedite bearing degradation. To validate the generalizability of the proposed model, additional comparative experiments are performed using datasets from Case Western Reserve University (CWRU) [27], the Society for Machinery Failure Prevention Technology (MFPT) [28], and Paderborn University (PU) [29]. Their parameters are summarized in Table 3.

**Figure 9:**
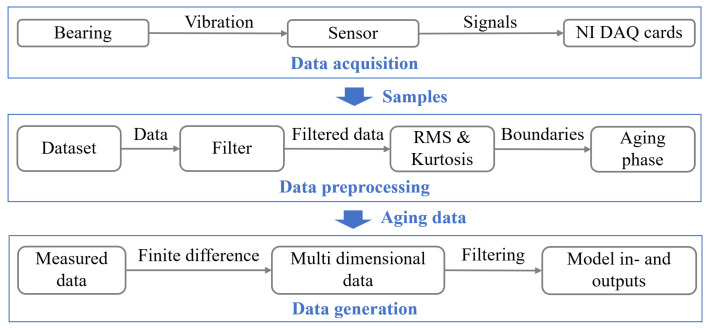
Data processing (aging data refers to the bearing data in the aging stage).

Figure 9 illustrates the data flow from sensor acquisition to modeling. During degradation experiments, data from the bearing’s healthy state throughout most of its lifecycle are not essential for the study. Modeling these stable acceleration responses is less challenging but consumes considerable computational resources. Therefore, we focus on the aging stage during data preprocessing. Acceleration signals are filtered using a one-dimensional stationary wavelet transform with Wavelet Symlets 8 and a frequency resolution of 50 Hz. The boundaries between the healthy, aging, and failure stages of the bearing are determined using the 3σ method. The division between the healthy and aging stages is completed by calculating the kurtosis value, while the starting point of the failure stage is determined by referring to the Root Mean Square (RMS). After segmenting the data from the bearing’s aging stage, finite difference is performed to generate multi-dimensional signals, filtered using a median filter with a window size of five to remove impulse noise from the finite difference process. These filtered finite difference data are used as inputs for the virtual state-space and further applied for model identification.

## 4. Experimental Results

This section presents sets of experiments to explore the effectiveness of the HW model based on virtual state-space and the impact of its different configurations. Critical configurations for HW modeling include the input and output structures of the state-space, their corresponding nonlinear estimators, and the zeros and poles of the transfer function. HW modeling is implemented using MATLAB’s System Identification Toolbox, with the Normalized Root Mean Square Error (NRMSE) as the criterion for evaluating modeling accuracy. NRMSE measures the fit between the model’s response and the actual data, expressed as a percentage (Modeling Accuracy = 100(1 − NRMSE)). The time required for modeling under various configurations is recorded to assess modeling efficiency. All experiments are conducted on a consistent software and hardware platform, and time measurements are averaged over three trials. The transfer function’s delay in the linear subsystem is set to one. Unless specified otherwise, all experiments use 126 samples collected from Bearing $3_{3}$ during the aging phase.

### 4.1. Model In- and Output

Initially, the composition of the model’s inputs and outputs needs to be determined. Single-input and single-output models are limited in their configurability and cannot simultaneously consider operating conditions and measurements, so they are not included in this study. Additionally, due to potential discrepancies between differentiation and finite difference data affecting HW model stability, particularly in complex Multi-Input-Multi-Output (MIMO) scenarios, the study focuses exclusively on Multi-Input-Single-Output (MISO) models.

Two primary configurations for the model inputs are examined. The first type includes $a_{y}$ and its multiple-order derivatives: $u=[a_{y},\dot{a}y,\ldots ,ay^{\left(n\right)}]$, where $n\in N^{+}$. The second type incorporates operating conditions to the first type, such as rotational speed $n_{r}$ and torque $T_{N}$, in addition to the derivatives: $u=[n_{r},T_{N},a_{y},\dot{a}y,\ldots ,ay^{\left(n\right)}]$. The study constructs a Multi-Input-Single-Output-Hammerstein–Wiener (MISO-HW) model based on the state-space representations in Equations (13) and (14) and evaluates the impact of four basic input–output compositions on modeling accuracy, as shown in Table 4. The linear module’s transfer function is configured with three poles and zero zeros. The experiments use the same nonlinear estimator for input and output modules, and various nonlinear estimators are compared for modeling accuracy, as shown in Table 5.

Results indicate that the modeling accuracy is significantly low when the input data’s order is lower than the output data’s. Therefore, Type 1 and Type 2 are not suitable for HW modeling. In Type 4, when using Unit Gain as the nonlinear estimator, the model accuracy reaches 98.70%. However, due to mathematical constraints, this configuration results in computational errors when validating modeling on other bearings. Similar errors occur in Type 2 as well because using finite differences instead of differentiation during data generation results in errors in the calculation process of a particular sample, leading to poor model stability. Therefore, we choose Type 3 as the input configuration, where the model input can be multiple variables of higher order than the output while disregarding operating conditions.

With the input data composition established, the study explores the HW model’s capacity to capture dynamic characteristics across different dimensions by varying input orders. It is assessed by modeling accuracy. With the experimental configuration and sample data constant, Unit Gain (UG) and One-Dimensional Polynomial (1-DP) are utilized as nonlinear input and output modules, and first through tenth-order derivatives of the acceleration signal serve as inputs. The original acceleration signal is used as the output. The resulting experimental data are presented in Table 6. The highest modeling accuracy is achieved when using three inputs with second-order data. However, increasing the order of inputs beyond the second order does not improve the model fitting results. Instead, it leads to decreased modeling efficiency due to increased computation time.

### 4.2. Nonlinear Module Estimator

Since the input–output configuration is determined, this set of experiments focus on selecting appropriate in- and output estimators to match the bearing’s nonlinear characteristics according to model fitting accuracy. The experiments use the optimal input–output configuration from Table 6, varying only the estimators. Due to numerous combinations, the same nonlinear estimator is initially applied to both input and output modules, as shown in the first group of experiments in Table 7. Results reveal that 1-DP and UG estimators achieve higher modeling accuracy, exceeding 87%. These findings suggest that these estimators better align with the nonlinear characteristics of the MISO-HW model’s input and output modules.

Consequently, UG is employed for input and output modules, as demonstrated in the second and third groups of experiments shown in Table 7. With the UG-1-DP combination, the modeling accuracy achieves a peak of 97.8659%, while the modeling time is 181.21 s. This time is slightly longer than the 134.87 s required for the 1-DP-UG combination, which, despite a lower accuracy improvement of 18.8475%, demonstrates superior efficiency. Further tests are conducted to assess the performance of the 1-DP combined with other estimators. Although the SN-1-DP combination yielded an accuracy of 96.0861%, its modeling time is significantly longer at 445.57 s, 2.46 times that of the UG-1-DP combination. This comparison indicates that the MISO-HW model performs optimally, with UG and 1-DP as input and output nonlinear estimators.

### 4.3. Zeros and Poles of Transfer Function

After identifying the optimal configuration for the nonlinear modules, further experimentation focuses on optimizing the model by adjusting the linear module settings. Various experiments are conducted to explore the impact of altering the number of zeros and poles in the transfer function of the HW model’s linear module. As per the requirement that a physically feasible control system must have more poles than zeros, the experimental design is outlined in Table 8. All configurations, except for the number of zeros and poles, use previously determined optimal values. In the first test group, increasing the number of poles improves modeling accuracy at first, reaching its peak with three poles before fluctuating downwards. Models with more than one zero exhibit instability, and all models with more than three zeros could not be constructed stably. Thus, it is inferred that the HW model is more stable when configured with no zeros. Both linear modules with three or eight poles achieve fitting accuracy exceeding 97.5% with a negligible computation time difference of 4.68 s. Therefore, the configuration with no zeros and three poles can be used for the highest modeling accuracy.

### 4.4. Analysis

Analysis of the four sets of experimental results reveals significant differences in how various parameters affect model accuracy. Firstly, the setting of zeros and poles in the linear module directly influences modeling stability. Based on the results in Table 8, configuring the transfer function of the linear module with no zeros allows the MISO-HW model to more accurately capture the linear characteristics of bearing dynamics. Secondly, the input–output configuration is crucial for achieving high model precision. Thirdly, among nonlinear modules, the choice of estimator impacts modeling accuracy, though to a lesser extent than the input–output configuration. Fourthly, the input data dimension has a relatively minor effect on accuracy, ranging from 2.7%.

The experiments in Table 6 show that accuracy did not improve with an increasing number of inputs. However, suppose the modeling accuracy increases with the number of inputs. In that case, it suggests that higher dimensions of data and model lead to high accuracy and better performance, which is different from the expected conclusion. This set of experiments focuses on the model dimension, aiming to identify model dimensions that enhance modeling performance. The additional input between different experiments is higher-dimensional data generated based on the virtual state theory. Thus, increasing the number of inputs expands the model dimension and reconstructs the mapping relationship instead of simply adding data. For example, the modeling accuracy declines when three-order derivative data are utilized, as shown in Table 6. This decline is attributed to the relatively simple system structure, where three dimensions suffice to effectively describe the dynamic characteristics of the bearing’s steady-state operation. Capturing the system’s dynamic feature in higher dimensions is more challenging, resulting in less contribution of higher dimensions to the modeling performance, thereby reducing the accuracy of the entire model.

The correctness of experimental results in Table 7 can be supported by the analysis of the bearing 5-DoF model. First, the 1-DP in the nonlinearity block of the HW model can be expressed by Equation (22):

(22) $$f_{p}\left(x\left(t\right)\right)=c\left(1\right){\left(x\left(t\right)\right)}^{n_{p}}+c\left(2\right){\left(x\left(t\right)\right)}^{n_{p}-1}+\ldots +c\left(n_{p}\right)x\left(t\right)+c\left(n_{p}+1\right),$$

where $c(n_{p})$ denotes the polynomial coefficients for the $n_{p}$-th term. Second, according to the Hertz contact theory, Equations (6)–(8) reveal that the acceleration $a_{y}$ is positively correlated with the 1-DP of deformation ($\delta_{j}^{1.5}$) and UG composed of other parameters ($k_{b},$$\gamma_{j}$, $\sin\phi_{j}$). By observing the similarity of these functional forms, we can conclude that it is mathematically interpretable to generate a high-precision bearing model using the 1-DP as a nonlinear module.

## 5. Verification

### 5.1. Validation of Optimized Configuration

Summarizing the comprehensive experimental results, the optimal configuration of the MISO-HW bearing model includes the transfer function with no zeros and three poles in the linear module, estimators employing UG and 1-DP in input and output nonlinear modules, and $u=[a_{y}^{\left(n+1\right)};a_{y}^{\left(n+2\right)};a_{y}^{\left(n+3\right)}]$ and $y=a_{y}^{\left(n\right)}$ as input–output type. Under this configuration, samples from Bearing 3_3 are used to construct 0th to ninth-order virtual state-space models. Herein, the original acceleration signal and its 0th to ninth-order derivatives are used as the outputs for the ten virtual state-space models.

**Figure 10:**
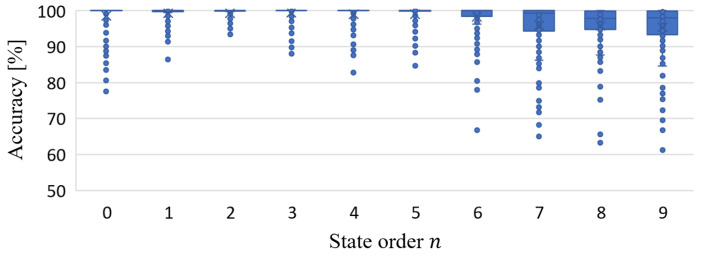
Modeling accuracy of Bearing 3_3.

After excluding outliers using the three-standard deviation method, the precision of the MISO-HW model constructed for Bearing $3_{3}$ consistently exceeds 90%. As detailed in Table 9 corresponding to Figure 10, the average precision across all state orders surpasses 93.72%, with relatively small standard deviations. Notably, for virtual state-space models with orders less than three, the modeling precision exceeds 98.29%, with a standard deviation within 3.64. This suggests that lower-order virtual state-space models may better capture the system’s dynamics. In conclusion, this modeling approach proves to be effective for Bearing $3_{3}$.

**Figure 11:**
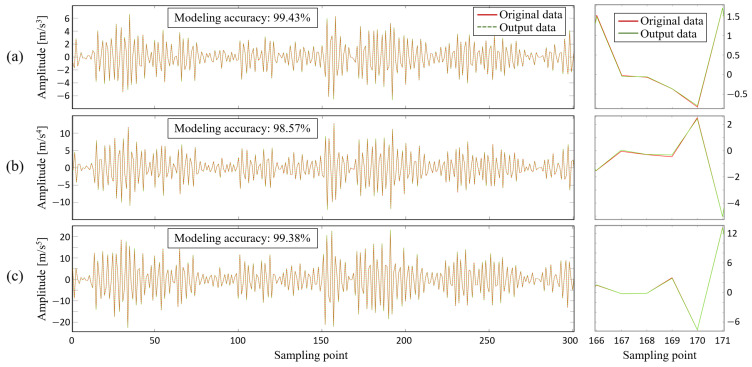
Comparison of raw and output signals of HW model for Bearing 3_3: (**a**) *n* = 1, (**b**) *n* = 2, (**c**) *n* = 3.

**Figure A1:**
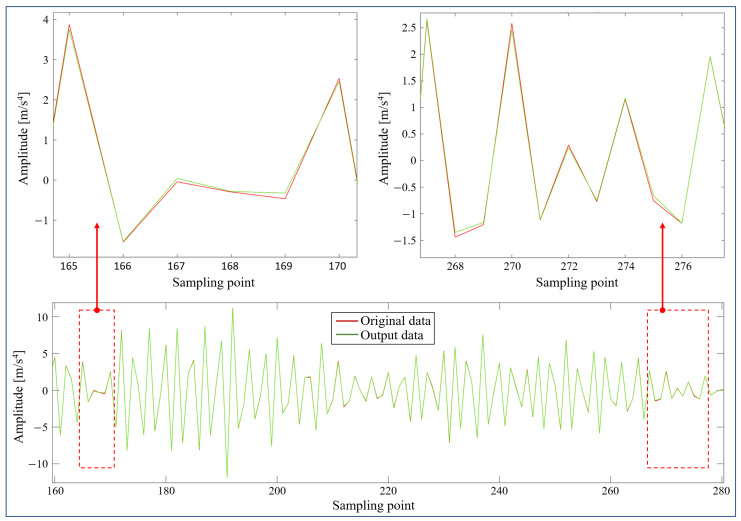
Signal comparison of HW model for the last sample of Bearing 3_3.

As a supplementary analysis, Figure 11 compares the original signal (in red) from the virtual state-space model for a single sample of Bearing $3_{3}$ with the HW model output signal (in green). The right side of the figure provides an enlarged comparison of six sampling points, where the high modeling accuracy results in a significant overlap between the two curves. Therefore, additional comparison plots with greater precision are presented in the Appendix A, as shown in Figure A1. The nearly identical trends and values of both signals further validate the efficacy of this modeling approach.

### 5.2. Verification with Bearings under Other Conditions

**Figure 12:**
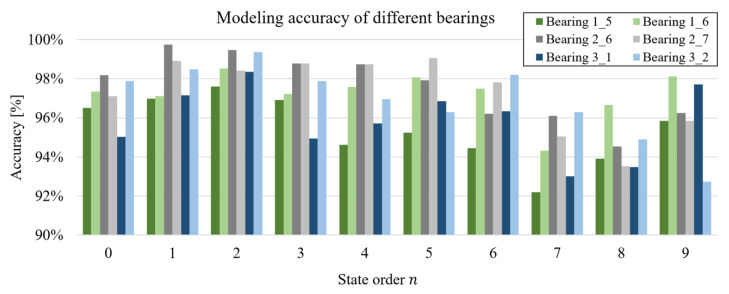
Modeling accuracy of bearings under different conditions.

**Figure 13:**
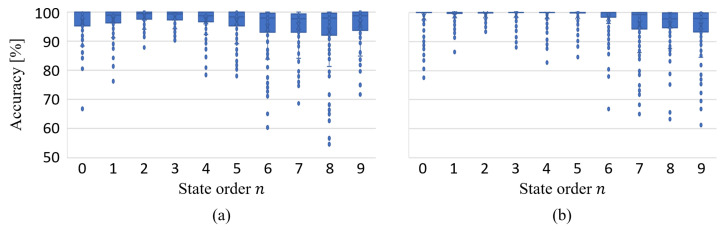
Modeling accuracy: (**a**) Bearing 1_5 under Condition 1, (**b**) Bearing 2_7 under Condition 2.

Following these initial findings, it is crucial to validate the proposed method across different bearings and operating conditions. With the same modeling configuration, experiments are conducted on 1700 samples from 17 bearings under all working conditions listed in Table 2. Figure 12 presents the experimental results for two bearings under each condition, with additional numerical results available in Table A1 in the Appendix A. The results indicate that all modeling accuracies exceed 90%, with an average accuracy of 96.30%. For instance, Figure 13 illustrates the modeling results for two bearings under varying operating conditions. Box plots, obtained after removing outliers, show that the average modeling accuracy for all bearing samples exceeds 95% under the NRMSE standard, with most samples achieving over 92% accuracy. Bearings under Condition 2 demonstrate an average accuracy exceeding 99% for virtual state-space models up to the fifth order. Thus, the MISO-HW model based on virtual state-space is highly effective for bearing fault modeling and exhibits robust generalization capabilities.

### 5.3. Verification with Other Datasets

**Figure 14:**
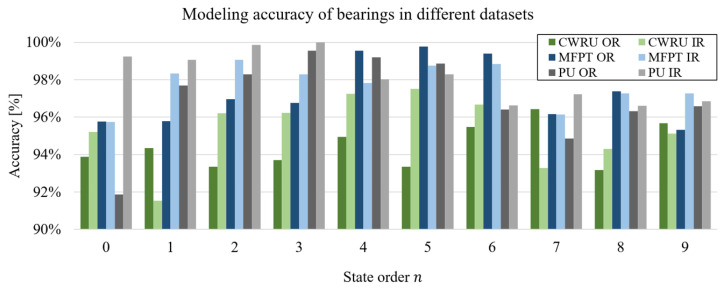
Modeling accuracy of bearings with faults in Outer Race (OR) and Inner Race (IR) in different datasets.

Comparative experiments using the CWRU, MFPT, and PU datasets are presented in Figure 14. Two types of bearing fault samples from each dataset are modeled with state orders ranging from 0 to 9. The number of sampling points for each bearing sample ranges from 100,000 to 120,000, with relevant parameters detailed in Table 3. The modeling accuracy for the six bearings across the three datasets consistently exceeds 90% in all state orders, with average accuracies of 94.44%, 95.34%, 97.28%, 97.76%, 96.96%, and 98.18%, respectively. The location of the bearing fault does not significantly impact the modeling accuracy. The modeling accuracy of the two CWRU samples (12.0 kHz) is lower than that of other bearing datasets (48.8 kHz and 64.0 kHz), presumably due to differences in data quality. The sampling frequency of these two bearings is lower than that of the other samples, and given that the data acquisition predates the other datasets by more than a decade, there may be gaps in equipment performance. Nonetheless, the high modeling accuracy of bearing samples from different datasets verifies the effectiveness of the proposed method and demonstrates its universality and generalizability.

### 5.4. Discussion

The first three orders depicted in Figure 12 reveal that the average modeling accuracy for the three bearings under Condition 3 is 96.84%, 98.57%, and 97.32%, respectively. For the 17 bearings overall, the average modeling accuracy exceeds 97.26%. These results further substantiate the effectiveness of this method in object transfer. Additionally, when assessing modeling accuracy across various working conditions, the average accuracy for the first three orders are 96.66%, 98.25%, and 97.57%, respectively. The MISO-HW modeling method, optimized with bearing samples from Condition 1, demonstrates robust performance across bearings in Conditions 2 and 3, underscoring the efficacy of this approach in transferring across working conditions.

Considering model dimensionality in Figure 14, the highest accuracy for each bearing is predominantly achieved at state orders 4 and 6. However, in Figure 12, the peak accuracy is concentrated at state orders 0 to 2. The data in Figure 12 are derived from healthy bearings in the aging stage, while Figure 14 reflects data from faulty bearings. This distinction suggests that the bearings in Figure 14 exhibit more significant dynamic nonlinearity and signal complexity. Thus, a higher-dimensional model is necessary to accurately capture these bearing faults’ dynamic characteristics, reinforcing the research value and motivation behind the proposed method.

## 6. Conclusions

This study presents a bearing fault modeling method based on virtual states and the HW model. The approach involves analyzing the 5-DoF dynamic model of bearings to identify suitable virtual states and input–output configurations, which are then used to establish virtual state-space models. Bearing vibration signals collected by acceleration sensors under various working conditions are employed in the experiments. Extensive testing of HW modeling configurations result in highly accurate bearing dynamics models, validating the effectiveness of the proposed method. The key contributions of this work can be summarized as follows:

1. The concept of virtual states is introduced, highlighting its significance in exploring system dynamic characteristics from multiple dimensions. This study employs virtual states and multi-dimensional bearing state-space models derived from measurable states in the dynamic analysis of bearings.
2. A bearing fault modeling method is developed using virtual state-space and HW models, utilizing acceleration signals and their multi-order derivatives to construct high-precision MISO-HW models based on virtual states.

The virtual state theory offers a theoretical framework for describing the dynamic characteristics of systems across various scales and dimensions, providing new insights for system modeling, identification, and fault diagnosis. While the proposed method shows great potential in PHM, it also presents certain limitations and areas for improvement. For instance, this method is currently restricted to MISO models due to the use of finite difference rather than differentiation in data generation, which compromises data quality and fails to meet the stability requirements of MATLAB’s built-in algorithms during the modeling process. Based on these findings, the following research directions are proposed:

1. To optimize data generation and improve the quality of high-dimensional data, derivative estimators could be developed, enabling the construction of MIMO models. These MIMO models allow for the extraction of characteristic parameters that more effectively represent the dynamic degradation processes in multidimensional models.
2. The state matrix $A_{n}$ in the models (refer to Figure 4) captures the relationships among states and the system’s dynamic characteristics across different dimensions. This matrix allows for the extraction of characteristic parameters highly correlated with bearing aging and faults, facilitating the development of high-precision diagnostic and prognostic methods.
3. This method can be further applied to different data sets and objects. A test bench equipped with a bearing test module and high-quality data acquisition terminals has been constructed. Experiments are planned to create a comprehensive dataset to validate the proposed method further. Furthermore, gear and rotor data will be employed to test this method following the design of a new test bench and relevant experiments.
4. Future improvements in the accuracy of this modeling method could be achieved by incorporating varying friction states. For example, integrating a friction coefficient that adapts to different friction conditions could enhance the model’s precision. Additionally, adopting an improved elastohydrodynamic lubrication modeling approach could serve as a superior alternative to the current Hertz contact theory-based modeling.

## Figures and Tables

**Table 1:** Parameters of bearings.

Parameter	Value
Diameter of the outer race $D_{or}$	29.1 mm
Diameter of inner race $D_{ir}$	22.1 mm
Bearing mean diameter $D_{m}$	25.6 mm
Inside diameter $D_{i}$	20.0 mm
Outside race diameter $D_{o}$	32.0 mm
Diameter of rolling elements $D_{b}$	3.5 mm
Thickness	7.0 mm
Number of rolling elements $n_{b}$	13

**Table 2:** Dataset of IEEE PHM 2012 prognostic challenge [26].

Operating Condition	Condition 1	Condition 2	Condition 3
Learning set	Bearing1_1	Bearing2_1	Bearing3_1
	Bearing1_2	Bearing2_2	Bearing3_2
Test set	Bearing1_3	Bearing2_3	Bearing3_3
	Bearing1_4	Bearing2_4	
	Bearing1_5	Bearing2_5	
	Bearing1_6	Bearing2_6	
	Bearing1_7	Bearing2_7	
Rotational speed [rpm]	1800	1650	1500
Force [N]	4000	4200	5000

**Table 3:** Spectrum error in ablation experiment results.

Dataset	CWRU	MFPT	PU	PHM Challenge
Number of rolling elements	9	8	8	13
Rolling element diameter [mm]	7.94	0.235	6.75	3.5
Pitch diameter [mm]	39.04	1.245	28.55	25.6
Sample rate [kHz]	12.0	48.8	64.0	25.6

**Table 4:** Configurations of the input and output.

Configuration	Type 1	Type 2	Type 3	Type 4
Input	$a_{y},\;{\dot{a}}_{y},\;{\ddot{a}}_{y}$	$a_{y},\;n_{r},\;T_{N}$	${\dot{a}}_{y},\;{\ddot{a}}_{y},\;{\dddot{a}}_{y}$	${\dot{a}}_{y},\;n_{r},\;T_{N}$
Output	${\dddot{a}}_{y}$	${\dot{a}}_{y}$	$a_{y}$	$a_{y}$

**Table 5:** Modeling accuracy with different configurations of the input and output.

Nonlinearity Estimator	Type 1	Type 2	Type 3	Type 4
Piecewise Linear function (PWL)	28.48%	7.97%	53.49%	76.74%
Sigmoid Network (SN)	29.46%	3.39%	68.19%	64.86%
Wavelet Network (WN)	29.12%	9.22%	77.60%	14.53%
Saturation (Sat)	34.24%	−11.88%	71.93%	8.67%
Dead Zone (DZ)	21.06%	17.17%	−66.33%	−27.16%
One-Dimensional Polynomial (1-DP)	21.32%	1.41%	94.95%	0.12%
Unit Gain (UG)	12.06%	−11.34%	86.91%	98.70%

**Table 6:** Configuration experiments with different input orders.

Input Order	Output	Inputs	Accuracy [%]	Duration [s]	Input Order	Output	Inputs	Accuracy [%]	Duration [s]
0	$a_{y}$	${\dot{a}}_{y}$	95.1475	43.42	5	$a_{y}$	${\dot{a}}_{y},{\ddot{a}}_{y},\ldots ,a_{y}^{\left(6\right)}$	96.3879	243.65
1	$a_{y}$	${\dot{a}}_{y},{\ddot{a}}_{y}$	95.4515	92.31	6	$a_{y}$	${\dot{a}}_{y},{\ddot{a}}_{y},\ldots ,a_{y}^{\left(7\right)}$	95.4982	270.04
2	$a_{y}$	${\dot{a}}_{y},{\ddot{a}}_{y},{\dddot{a}}_{y}$	97.8659	182.01	6	$a_{y}$	${\dot{a}}_{y},{\ddot{a}}_{y},\ldots ,a_{y}^{\left(7\right)}$	95.4982	270.04
3	$a_{y}$	${\dot{a}}_{y},{\ddot{a}}_{y},\ldots ,a_{y}^{\left(4\right)}$	95.5293	185.62	8	$a_{y}$	${\dot{a}}_{y},{\ddot{a}}_{y},\ldots ,a_{y}^{\left(9\right)}$	96.7953	323.06
4	$a_{y}$	${\dot{a}}_{y},{\ddot{a}}_{y},\ldots ,a_{y}^{\left(5\right)}$	95.6098	222.27	9	$a_{y}$	${\dot{a}}_{y},{\ddot{a}}_{y},\ldots ,a_{y}^{\left(10\right)}$	95.8023	361.37

**Table 7:** Modeling accuracy with different nonlinearity estimators.

Input Module	Output Module	Accuracy [%]	Duration [s]	Input Module	Output Module	Accuracy [%]	Duration [s]
PWL	PWL	57.0940	320.01	PWL	UG	96.2595	230.29
SN	SN	71.8959	515.63	SN		96.7476	305.78
WN	WN	31.5333	530.03	WN		95.1923	298.02
Sat	Sat	74.4528	253.84	Sat		86.6872	209.66
DZ	DZ	−165.9393	312.46	DZ		77.6185	263.64
1-DP	1-DP	94.2116	238.85	1-DP		97.8659	181.21
UG	UG	87.9686	157.12		UG	94.5437	250.42
1-DP	PWL	38.9662	243.43		SN	95.0212	398.24
	SN	20.4830	349.05	UG	WN	57.3083	308.79
	WN	29.2596	341.45		Sat	76.0919	229.48
	Sat	54.7752	184.98		DZ	−308.0773	224.81
	DZ	−20.0300	225.96		1-DP	79.0184	134.87
PWL	1-DP	95.4379	253.76				
SN		96.0861	445.57				
WN		86.2918	365.10				
Sat		78.9940	256.11				

**Table 8:** Modeling accuracy with different configurations of zeros and poles.

Test Group	Zeros Number	Poles Number	Accuracy [%]	Duration [s]	Test Group	Zeros Number	Poles Number	Accuracy [%]	Duration [s]
Group 1	0	1	95.7328	96.14	Group 2	1	1	87.1260	164.44
	0	2	96.2157	125.48		1	2	85.8251	207.27
	0	3	97.8659	182.21		1	3	56.5755	230.76
	0	4	96.5741	154.12		1	6	65.8701	234.77
	0	5	95.8959	163.29		1	4, 5, 7, 8, 9	\	\
	0	6	96.8135	186.20	Group 3	2	2	85.8871	191.79
	0	7	95.4290	179.67		2	3, 4, …, 9	\	\
	0	8	97.5603	177.53	Group 4	3	3	0.0272	369.05
	0	9	96.5667	183.86		3	4, 5, …, 9	\	\
	0	10	94.3603	192.23	Group 5	4	4, 5, …, 9	\	\

**Table 9:** Accuracy parameters of Bearing 3_3.

State Order *n*	0	1	2	3	4	5	6	7	8	9
Min. accuracy [%]	81.63	89.92	84.96	64.01	46.76	74.95	67.11	59.31	58.67	60.10
Max. accuracy [%]	100.00	100.00	100.00	100.00	100.00	100.00	100.00	100.00	100.00	100.00
Mean accuracy [%]	98.58	98.98	98.29	96.21	95.34	96.44	94.89	93.73	93.72	94.92
Standard deviation	3.64	2.14	3.03	6.38	8.56	5.24	6.59	7.88	8.27	8.03

## Data Availability

The raw data supporting the conclusions of this article will be made available by the authors on request.

## Appendix A

**Table A1:** Accuracy of bearings under different working conditions [%].

State Order *n*	0	1	2	3	4	5	6	7	8	9
Bearing 1_1	95.32	95.65	96.51	94.89	94.19	96.10	97.17	94.85	94.03	96.70
Bearing 1_2	94.26	95.81	96.07	96.92	97.79	95.78	96.06	95.74	96.42	97.37
Bearing 1_3	96.44	96.41	96.04	96.42	97.21	97.87	97.94	97.79	97.02	97.38
Bearing 1_4	95.86	95.22	96.29	96.29	94.50	92.41	93.44	94.54	95.42	95.91
Bearing 1_5	96.50	96.97	97.59	96.92	94.61	95.23	94.44	92.19	93.91	95.84
Bearing 1_6	97.35	97.10	98.52	97.22	97.58	98.07	97.49	94.32	96.65	98.12
Bearing 1_7	98.66	97.96	99.27	98.47	98.97	97.77	93.31	95.05	91.77	95.32
Bearing 2_1	99.75	98.17	94.96	92.30	94.32	91.84	90.25	93.99	90.82	93.87
Bearing 2_2	99.58	98.94	98.66	97.70	94.99	93.82	93.17	94.14	91.48	95.57
Bearing 2_3	98.33	98.14	98.81	97.41	95.91	97.25	97.82	95.21	95.78	96.17
Bearing 2_4	98.28	99.38	98.79	97.68	97.49	97.06	94.01	96.36	96.18	96.11
Bearing 2_5	97.00	96.83	95.84	97.71	98.06	98.14	97.57	95.49	96.24	97.66
Bearing 2_6	98.18	99.74	99.46	98.77	98.73	97.93	96.20	96.09	94.53	96.24
Bearing 2_7	97.11	98.91	98.41	98.79	98.74	99.05	97.81	95.05	93.52	95.85
Bearing 3_1	95.03	97.14	98.34	94.93	95.72	96.84	96.33	93.00	93.48	97.70
Bearing 3_2	97.88	98.48	99.35	97.88	96.95	96.30	98.20	96.29	94.90	92.74
Bearing 3_3	97.91	97.63	96.41	94.47	94.05	94.16	94.04	93.55	94.30	95.80

## References

[1] Rai A., Upadhyay S.H. (2016). A review on signal processing techniques utilized in the fault diagnosis of rolling element bearings. Tribol. Int..

[2] Jalan A.K., Mohanty A. (2009). Model based fault diagnosis of a rotor–bearing system for misalignment and unbalance under steady-state condition. J. Sound Vib..

[3] Mishra C., Samantaray A., Chakraborty G. (2017). Ball bearing defect models: A study of simulated and experimental fault signatures. J. Sound Vib..

[4] Xi S., Cao H., Chen X., Niu L. (2018). Dynamic modeling of machine tool spindle bearing system and model based diagnosis of bearing fault caused by collision. Procedia CIRP.

[5] Javanmardi D., Rezvani M.A. (2023). Rail vehicle axlebox roller bearing life and failure analysis based on the Hertz contact theory, finite element modeling, and field observations. World J. Eng..

[6] Dharap G., Talbot D. (2023). A Quasi-Static Load Distribution Model for Deep Groove Ball Bearings. Proceedings of the International Design Engineering Technical Conferences and Computers and Information in Engineering Conference.

[7] Qian L., Pan Q., Lv Y., Zhao X. (2022). Fault detection of bearing by resnet classifier with model-based data augmentation. Machines.

[8] Janssens O., Slavkovikj V., Vervisch B., Stockman K., Loccufier M., Verstockt S., Van de Walle R., Van Hoecke S. (2016). Convolutional neural network based fault detection for rotating machinery. J. Sound Vib..

[9] Gilbert Chandra D., Srinivasulu Reddy U., Uma G., Umapathy M. (2023). Group normalization-based 2D-convolutional neural network for intelligent bearing fault diagnosis. J. Braz. Soc. Mech. Sci. Eng..

[10] Li F., Wang L., Wang D., Wu J., Zhao H. (2023). An adaptive multiscale fully convolutional network for bearing fault diagnosis under noisy environments. Measurement.

[11] Yu Z., Zhang C., Liu J., Deng C. (2023). SKND-TSACNN: A novel time-scale adaptive CNN framework for fault diagnosis of rotating machinery. Knowl.-Based Syst..

[12] Keshun Y., Puzhou W., Yingkui G. (2024). Towards efficient and interpretative rolling bearing fault diagnosis via quadratic neural network With Bi-LSTM. IEEE Internet Things J..

[13] Ruan D., Wu Y., Yan J. Remaining useful life prediction for aero-engine based on lstm and cnn. Proceedings of the 2021 33rd Chinese Control and Decision Conference (CCDC).

[14] Pan Y., Wang H., Chen J., Hong R. (2023). Fault recognition of large-size low-speed slewing bearing based on improved deep belief network. J. Vib. Control.

[15] Song X., Lyu X., Sun S., Li C. (2023). A novel deep learning model for fault diagnosis of rolling-element bearing based on convolution neural network and recurrent neural network. Proc. Inst. Mech. Eng. Part E J. Process. Mech. Eng..

[16] Li Z., Wang J., Ruan D., Yan J., Gühmann C. (2023). Bearing Digital Twin Based on Response Model and Reinforcement Learning. Lubricants.

[17] Tian H., Fan H., Feng M., Cao R., Li D. (2023). Fault diagnosis of rolling bearing based on hpso algorithm optimized cnn-lstm neural network. Sensors.

[18] Wang Z., Liu Z., Xiao J. (2023). A Complete Modeling Method for Nonlinear Dynamic Processes Based on Wiener Structured Neural Network and Wiener-Hammerstein Structured Neural Network. Chin. Control. Instrum. Chem. Ind..

[19] Zhong G., Yu Q., Wang L. (2023). Iterative learning algorithm with forgetting factor for Hammerstein-Wiener time-varying systems. Chin. High Technol. Lett..

[20] Cui L., Chen X., Chen S. (2015). Dynamics modeling and analysis of local fault of rolling element bearing. Adv. Mech. Eng..

[21] Ruan D., Chen Y., Gühmann C., Yan J., Li Z. (2022). Dynamics Modeling of Bearing with Defect in Modelica and Application in Direct Transfer Learning from Simulation to Test Bench for Bearing Fault Diagnosis. Electronics.

[22] Li Z. (2023). Data-Driven Bearing Fault Modeling and Its Application in Bearing Fault Diagnosis. Master’s Thesis.

[23] Li Y., Chen X., Mao Z. (2017). Model predictive control synthesis algorithm based on polytopic terminal region for Hammerstein-Wiener nonlinear systems. Chin. J. Cent. South Univ..

[24] Smith W.A., Randall R.B. (2016). Cepstrum-based operational modal analysis revisited: A discussion on pole–zero models and the regeneration of frequency response functions. Mech. Syst. Signal Process..

[25] Wills A., Schön T.B., Ljung L., Ninness B. (2013). Identification of hammerstein–wiener models. Automatica.

[26] Nectoux P., Gouriveau R., Medjaher K., Ramasso E., Chebel-Morello B., Zerhouni N., Varnier C. PRONOSTIA: An experimental platform for bearings accelerated degradation tests. Proceedings of the IEEE International Conference on Prognostics and Health Management, PHM’12.

[27] Smith W.A., Randall R.B. (2015). Rolling element bearing diagnostics using the Case Western Reserve University data: A benchmark study. Mech. Syst. Signal Process..

[28] Condition Based Maintenance Fault Database for Testing of Diagnostic and Prognostics Algorithms. https://www.mfpt.org/fault-data-sets/.

[29] Bearing Data Center, KAt-Data Center, Paderborn University. http://groups.uni-paderborn.de/kat/BearingDataCenter/.

